# A Novel Modified Super-Twisting Control Augmented Feedback Linearization for Wearable Robotic Systems Using Time Delay Estimation

**DOI:** 10.3390/mi12060597

**Published:** 2021-05-21

**Authors:** Brahim Brahmi, Ibrahim El Bojairami, Tanvir Ahmed, Asif Al Zubayer Swapnil, Mohammad AssadUzZaman, Inga Wang, Erin McGonigle, Mohammad Habibur Rahman

**Affiliations:** 1 Mechanical Engineering Department, McGill University, Montreal, QC H3A 0G4, Canada; ibrahim.elbojairami@mail.mcgill.ca; 2 Biomedical Engineering Department, University of Wisconsin-Milwaukee, Milwaukee, WI 53211, USA; tanvir@uwm.edu (T.A.); aswapnil@uwm.edu (A.A.Z.S.); assaduz2@uwm.edu (M.A.); rahmanmh@uwm.edu (M.H.R.); 3 Rehabilitation Sciences and Technology, University of Wisconsin-Milwaukee, Milwaukee, WI 53211, USA; wang52@uwm.edu; 4 Physical Medicine & Rehabilitation Department, Medical College of Wisconsin (MCW), Wauwatosa, WI 53226, USA; emcgonigle@mcw.edu

**Keywords:** super-twisting control, time delay estimation, feedback linearization, uncertain dynamics

## Abstract

The research presents a novel controller designed for robotic systems subject to nonlinear uncertain dynamics and external disturbances. The control scheme is based on the modified super-twisting method, input/output feedback linearization, and time delay approach. In addition, to minimize the chattering phenomenon and ensure fast convergence to the selected sliding surface, a new reaching law has been integrated with the control law. The control scheme aims to provide high performance and enhanced accuracy via limiting the effects brought by the presence of uncertain dynamics. Stability analysis of the closed-loop system was conducted using a powerful Lyapunov function, showing finite time convergence of the system’s errors. Lastly, experiments shaping rehabilitation tasks, as performed by healthy subjects, demonstrated the controller’s efficiency given its uncertain nonlinear dynamics and the external disturbances involved.

## 1. Introduction

Survivors of strokes usually suffer paralysis and loss of physical strength, often on one side of the body, such as the upper extremity [1]. Upper-limb dysfunctions create difficulties in conducting daily activities such as eating, dressing, and cleaning, resulting in a significant influence on the victim’s everyday life [2]. In such cases, rehabilitation treatments are practiced to remedy lost functional capacity, gain new skills, and enhance the quality of life [1]. Those are usually rehab therapies consisting of a set of medical exercises guided by a therapist to increase the range of motion and muscle strength. Recently, a new method of rehabilitation has emerged, namely, robotic rehabilitation. This has attracted a lot of attention in the scientific community due to robots’ ability to supplement treatments provided by conventional physiotherapy. The importance of rehabilitation robots lies in their ability to provide intensive physiotherapy, for a prolonged period of time, regardless of the availability of a therapist [2].

Rehabilitation robots are novel devices designed to overcome conventional physiotherapy limitations, as well as create new, user-specific rehabilitation exercises [2,3,4]. Typically, such robots are designed in a way to adjust and be identical to human arm configurations [2]. For patients with upper-limb impairments, exoskeletons are usually worn on the upper-limb lateral side [5]. With multiple degrees of freedom (DOFs), the exoskeleton is capable of achieving several arm configurations in its workspace [3]. However, when a robot’s DOFs are increased, obtaining the robot’s dynamic model becomes arduous due to its complex mechanical structure, actuator intricacy, as well as other involved parameters such as nonlinear friction forces and backlash. This is not to mention that variation of subjects’ physiological conditions, such as nonlinear musculoskeletal system characteristics, upper limb weights, spasticity dystonia, and muscle weakness in neurological patients, contribute to uncertainty in exoskeleton robot’s dynamics and control. In other words, the manipulator is potentially subjected to both parametric uncertainties and unknown nonlinear functions [3]. Besides, the nonlinear uncertain dynamic model and external forces can transform to an unknown function, which in turn deteriorates a robot’s performance [3]. In this case, to better achieve the therapeutic activity, a solid adaptive controller becomes essential to estimate dynamic model uncertainties and external forces.

Uncertain nonlinear dynamics control is an interesting, highly challenging topic in the nonlinear control engineering field. A commonly used technique to resolve the problem of nonlinear decoupling control is Feedback Linearization (FL). FL aims to algebraically transform a nonlinear system to its equivalent linear one in order to apply the straightforward linear control theory. Despite this technique being applicable for some practical control problems, the presence of hard nonlinear parameters, and/or system uncertainties, prevents conventional linear control representations from appropriately describing the original system, which deteriorates a system’s performance and accuracy. On the other hand, numerous nonlinear control systems, such as the conventional adaptive control [6], $H_{\infty }$ control [7], and sliding mode control [6,8] with backstepping [3,4,9,10], have been designed to overcome the effects of uncertain nonlinear dynamics and unexpected external disturbances. That being said, sliding mode control (SMC) is considered one of the most robust nonlinear controllers developed to overcome uncertain dynamics. SMCs are fundamentally based on high switching gain values, forcing a system’s trajectory to converge to a selected sliding surface [6,8,9]. However, such high switching gains create “chattering”, a common problem in robotics that potentially damages robots’ actuators [11].

Numerous solutions have been proposed to reduce chatterings, such as the exponential reaching law controller [5], high-order sliding mode [12], super-twisting algorithm [13], and the modified super-twisting [14]. Involved intelligent sliding mode controllers were also proposed, whereby the fuzzy system and neural network are integrated with the sliding mode [15,16]. Nevertheless, such approaches involve heavy computations, making their implementation questionable. Regardless, the super-twisting control method has the potential to significantly reduce undesired chattering [17]. Yet, when system states are far from the desired sliding surface, the finite-time convergence to the selected surface cannot be guaranteed [18]. Furthermore, the super-twisting control law is based on the power rate reaching technique [19,20], which is potentially beneficial and practical for the design of structure variable control systems. Despite this law’s ability to reduce chattering [20,21], it still suffers from numerous shortcomings, with probably the most critical one being the power rate reaching law sensitivity to nonlinear modeling uncertainties [20,21]. That is, even if uncertainty dynamics satisfy the smooth and bounded matching condition with known boundaries [22,23,24,25,26], the system remains highly sensitive [20]. In addition, when the selected surface is close to zero, the time derivative of the desired surface also converges to zero, to which the control input becomes unable to intercept uncertain dynamics and unexpected disturbances anymore. Besides, defining uncertainty boundaries and external disturbances’ exact margins is a frustrating limitation of super-twisting control [22,23,24,25,26]. The overestimation of such boundaries causes higher control gains than ultimately desired [22,23,24,25,26].

In light of the challenges above, a new Modified Super-Twisting Augmented Feedback Linearization based on Time Delay Control (MSTFLTDC) is designed. The present research proposes the solution of transforming the nonlinear model to its equivalent linear, via input/output Feedback Linearization (FL). Thereafter, a nonlinear super-twisting control and Time Delay Estimation (TDE) are integrated to estimate a system’s hard nonlinearities, represented by uncertain nonlinear dynamics and external disturbances. TDE is employed due to its strong ability to significantly limit uncertainty effects [18,19]. This is achieved via employing a time-delayed knowledge from the previous state response and control input. Instead of any prior accurate knowledge of the exoskeleton robot parametric model, it rather estimates the uncertain dynamic model and external perturbations. In effect, the proposed controller, “MSTFLTDC”, provides increased robustness and enhanced accuracy, without being sensitive to involved uncertain dynamics and disturbances. In addition, to avoid the effect of uncertainty overestimation involved with super-twisting gains, a new exponential reaching law is utilized [5]. This law is considered an adaptation technique to potentially provide a fast system reaching response, increase control scheme reachability, and limit chattering—that is, the proposed control scheme aims to globally reduce the chattering problem. Lastly, the Lyapunov’s theory was implemented to potentially prove closed-loop form stability, whereby the asymptotic convergence of the output tracking errors would be ensured on finite time. The main contributions of this paper can be summarized in the following points:

1. In the feedback linearization approach adopted in this paper, the dynamic model of the robot is transformed into a simpler form. Based on this form, the control law is derived. By employing a suitable transformation to this control law, it becomes usable to the original physical system, while it is excellent for trajectory planning/following tasks.
2. Develop a control law based on a modified super-twisting controller with Time Delay Estimation (TDE) that supplies an approximation of uncertainties and external disturbances by using a step into the past of the inputs and the output of the system.
3. An adaptive exponential term function of the switching surface called exponential reaching law (ERL) is integrated with the proposed reaching law. The ERL presents a kind of adaptation of the switching gains. If the tracking error value becomes large, the switching gains become large too, such as a faster convergence during the reaching phase is realized. The switching gains become small, e.g., the phenomenon of chattering is reduced during the sliding phase.
4. Experimental studies conducted using a new exoskeleton robot named Smart Robotic Exoskeleton (SREx) to evaluate the proposed control scheme’s performance with respect to providing excellent tracking, small steady-state error, and reduced chattering.

The rest of the paper is organized as follows: Section 2 presents the manipulator’s dynamic characterization as well as the problem formulation. Section 3 demonstrates the proposed controller (MSTFLTDC) objective and a detailed description of the control strategy. Section 4 illustrates the modeling of an exoskeleton robot, named SREx, and provides its experimental results. In this section, the proposed controller is experimentally evaluated via healthy subjects upon implementing a designed trajectory tracking corresponding to a specific passive physical therapy. Finally, conclusions and future work are presented in Section 5.

## 2. Manipulator Robot Mathematical Characterization

### 2.1. Robot Modelling

The dynamics of a fully actuated manipulator robot with *n* DOFs can be expressed in joint space, using the Lagrangian approach, as follows:

(1) $$M\left(\theta \right)\ddot{\theta }+C\left(\theta ,\dot{\theta }\right)\dot{\theta }+G\left(\theta \right)+F\left(\theta ,\dot{\theta }\right)=\tau +\tau_{ex}$$

where $\theta ,\dot{\theta }$ and $\ddot{\theta }\in R^{n}$ are, respectively, the joints’ position, velocity, and acceleration vectors. $M\left(\theta \right)\in R^{n\times n}$ is the symmetric, positive definite, inertia matrix. $C\left(\theta ,\dot{\theta }\right)\dot{\theta }\in R^{n}$ represents the centrifugal and Coriolis effects. $G\left(\theta \right)\in R^{n}$ is the gravitational vector, τ is the torques vector, $\tau_{ex}$ is the external disturbances, and $F\left(\theta ,\dot{\theta }\right)\in R^{n}$ is the nonlinear friction vector. Without loss of generality, the robot system dynamic model can be rewritten as follows:

(2) $$\{\begin{array}{l} \;M\left(\theta \right)=M_{0}\left(\theta \right)+\Delta M\left(\theta \right)\\ C\left(\theta ,\dot{\theta }\right)=C_{0}\left(\theta ,\dot{\theta }\right)+\Delta C\left(\theta ,\dot{\theta }\right)\\ \;\;G\left(\theta \right)=G_{0}\left(\theta \right)+\Delta G\left(\theta \right) \end{array}$$

where $M_{0}\left(\theta \right),C_{0}\left(\theta ,\dot{\theta }\right)$ and $G_{0}\left(\theta \right)$ are, respectively, the known parts of the inertia matrix, Coriolis and centrifugal matrix, and gravity vector. The terms $\Delta M\left(\theta \right),\;\Delta C\left(\theta ,\dot{\theta }\right)$, and ΔG(θ) represent the uncertainties. Introducing the variable $x={\left[x_{1},x_{2}\right]}^{T}={\left[\theta ,\dot{\theta }\right]}^{T}\in R^{2n}$, the dynamic model, expressed by (1), can be rewritten in state representation as follows:

(3) $$\{\begin{array}{l} \dot{x}=B\left(x\right)u+F\left(x\right)+H\left(x\right)\\ y=P\left(x\right)=Cx \end{array}$$

where u=τ; y is the output vector and $C=\left[\begin{array}{cc} I_{n\times n} & 0_{n\times n} \end{array}\right]$ is the output matrix with $I_{n\times n}$ being the identity matrix. The vectors B(x), F(x), and H(x) are defined as follows:

$$B\left(x\right)=\left[\begin{array}{c} 0_{n\times n}\\ b\left(x\right) \end{array}\right];\;F\left(x\right)=\left[\begin{array}{c} x_{2}\\ f\left(x\right) \end{array}\right];\;H\left(x\right)=\left[\begin{array}{c} 0_{n\times 1}\\ h\left(x\right) \end{array}\right]$$

With $b\left(x\right)=M_{0}^{-1}\left(\theta \right)$ and it is assumed to be bounded and invertible matrix, $f\left(x\right)=M_{0}^{-1}\left(\theta \right)\left(-C_{0}\left(\theta ,\dot{\theta }\right)\dot{\theta }-G_{0}\left(\theta \right)\right)$, and $h\left(x\right)=M_{0}^{-1}\left(\theta \right)\left(\tau_{ex}-\Delta M\left(\theta \right)\ddot{\theta }-\Delta C\left(\theta ,\dot{\theta }\right)\dot{\theta }-\Delta G\left(\theta \right)-F\left(\theta ,\dot{\theta }\right)\right)$. For simplicity, let us denote f(x)=f(t) and h(x)=h(t), where, x is the variable related to time.

### 2.2. Robot Manipulator Input/Output Linearization

**Figure 1:**
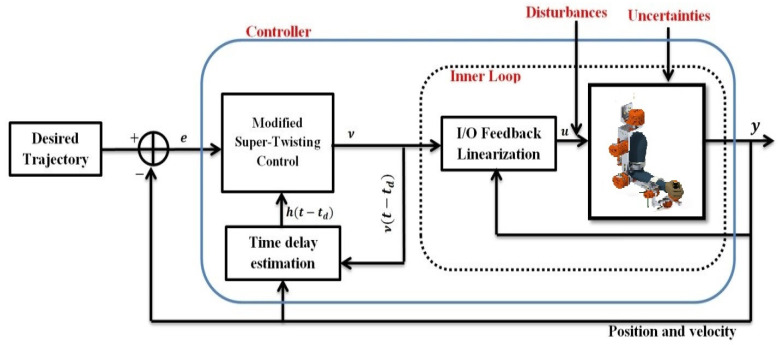
Block diagram of the proposed controller.

In order to investigate the system’s performance accuracy as well as finite time errors’ convergence in the presence of uncertainties and external perturbations, a new Modified super-twisting augmented feedback linearization with TDE is applied. For this purpose, the dynamic system is first linearized based on the one input/output feedback linearization approach. This is achieved via two loops: the first is an inner loop designed to realize the linearization of the system input/output state relation and build a nonlinear control law. The second is an outer loop aimed to control the linear system and realize closed-loop system stability [6,9,27]. The global structure of the control system is shown in Figure 1.

The objective of the input-output linearization technique is to obtain a direct relationship between the system output y and the control action input u. This is achieved via differentiating the output, y, r times (r being the relative degree) using Lie derivatives to obtain an expression between the system’s input and output. To better understand the linearization procedure, Lie derivatives are first introduced and defined.

**Definition** **1.** *Consider the system represented by Equation (3), with its vectors being of a relative degree* $\left[r_{1},\ldots .,r_{n}\right]$*. The relative degree is assigned for n subsystems such that:*

(4) $$y_{i}^{k}=L_{F}^{\left(k\right)}P_{i};for\;0\leq k\leq r_{i-1}\;y_{i}^{\left(r_{i}\right)}=L_{F}^{\left(r_{i}\right)}P_{i}+\overset{n}{\underset{j=1}{\sum }}L_{B_{j}}\left(L_{F}^{\left(r_{i}-1\right)}P_{i}\right)u_{i}$$

Applying the Lie derivative on the known part of system (3), in which case the relative degree r is 2, the output vector then becomes:

(5) $$\dot{y}=L_{F}P\left(x\right)=x_{2}\;\ddot{y}=L_{F}^{\left(k\right)}P\left(x\right)+L_{B}L_{F}P\left(x\right)u\;=f\left(x\right)+b\left(x\right)u$$

According to Equation (5), there is an explicit relation between the system’s input and output. Hence, the control input is chosen as:

(6) $$u=b{\left(x\right)}^{-1}\left(\nu -f\left(x\right)\right)$$

As observed in Equation (6), the input vector is controlled by an external parameter ν. In such a case, the relation between the novel control ν and the system output can be found as follows:

(7) $$\begin{array}{l} \ddot{y}=f\left(x\right)+b\left(x\right)u\\ \;\;=\nu \end{array}$$

From the proposed feedback linearization given by Equations (6) and (7), and comparing against the system Equation (3), it becomes straightforward to obtain the following system:

(8) $$\{\begin{array}{l} {\dot{x}}_{1}=x_{2}\\ {\dot{x}}_{2}=h\left(x\right)+\nu \end{array}$$

Denoting h(x)=h(t), Equation (8) can then be expressed by:

(9) $$\dot{z}=Az+B\left(h\left(x\right)+\nu \right)$$

where $z={\left[z_{1},z_{2}\right]}^{T}={\left[x_{1},x_{2}\right]}^{T}$, $A=\left[\begin{array}{cc} 0_{n\times n} & I_{n\times n}\\ 0_{n\times n} & 0_{n\times n} \end{array}\right]$, and $B=\left[\begin{array}{c} 0_{n\times n}\\ I_{n\times n} \end{array}\right]$.

### 2.3. Problem Formulation

The present research addresses the challenge of developing a robust adaptive controller capable of providing excellent trajectory tracking under an unknown robot dynamic model, the presence of uncertain nonlinear dynamics, and a system subjected to external disturbances. The objective is to further show finite time convergence of the dynamic errors to zero, as well as to provide a stability analysis under the following assumptions:

**Assumption 1:** Joint position and velocity are measurable.
**Assumption 2:** The matrix $M_{0}\left(\theta \right)$ is assumed to be bounded and invertible.
**Assumption 3:** The pseudo-Jacobian matrix is non-singular.
**Assumption 4:** Desired trajectory is bounded.
**Assumption 5:** The uncertain functions h(t) are continuously differentiable concerning the time variable and do not vary largely during a small $t_{s}$ period.
**Assumption 6:** The velocity and the acceleration outputs of the system are bounded.

### 2.4. Control Design

The control scheme aims to develop a robust adaptive controller able to provide satisfactory trajectory tracking even though the dynamic model of the robot is not completely known. Additionally, even under unknown uncertainty boundaries, the control system should be potentially insensitive to bound uncertain dynamics and external disturbances. Lastly, upon integrating a new reaching law, the controller aims to eliminate the chattering problem, as well as ensure system dynamic errors’ convergence to zero in a finite time.

The standard super-twisting control is given by:

(10) $$\{\begin{array}{l} {\dot{s}}_{i}=-\lambda_{1i}{\left(\left|s_{i}\right|\right)}^{\frac{1}{2}}sign\left(s_{i}\right)+w_{i}\\ {\dot{w}}_{i}=-\lambda_{2i}sign\left(s_{i}\right);\;i=1\ldots .n \end{array}$$

where $\lambda_{1i}>0;\;\lambda_{2i}>0$ are positive constants.

Prior to designing the proposed control scheme, consider $z^{d}\in R^{2n}$ and $z\in R^{2n}$ being the desired and measured trajectories, respectively, where $e=z-z^{d}$ and $\dot{e}=\dot{z}-{\dot{z}}^{d}$. The sliding surface is then selected as follows:

(11) $$s=\varphi \left[\dot{e}+\left[\begin{array}{c} I_{n\times n}\\ 0_{n\times n} \end{array}\right]e\right]$$

where $\varphi =\left[\begin{array}{cc} \rho I_{n\times n} & I_{n\times n} \end{array}\right]\in R^{n\times 2n}$ is a full row rank constant matrix, and ρ is a positive constant. It then follows that $\varphi B=I_{n\times n}$ and $\varphi \left[\begin{array}{c} I_{n\times n}\\ 0_{n\times n} \end{array}\right]=\rho I_{n\times n}$.

Taking the derivative of Equation (11) results in:

(12) $$\dot{s}=\varphi Az_{2}+h\left(x\right)+\nu -\varphi {\dot{z}}^{d}+\rho I_{n\times n}\dot{e}$$

**Theorem** **1.** *Consider the robot system described by Equation (8). To ensure: (1) the system’s global asymptotic stability, (2) chattering elimination, and (3) the finite-time convergence of the tracking errors to zero, a new adaptive controller using TDE is proposed as follows:*

(13) $$\nu =\lambda_{1}\left(s\right)sign\left(s\right)+\varphi {\dot{z}}^{d}-\varphi Az-\rho \dot{e}-h\left(t\right)-\int_{0}^{t}\lambda_{2}\left(s\right)sign\left(s\right)$$

where $\lambda_{1}\left(s\right)=diag\left(\frac{-\lambda_{1i}}{Q_{i}\left(s_{i}\right)}{\left(\left|s_{i}\right|\right)}^{\frac{1}{2}}\right)$, $sign\left(s\right)={\left[sign\left(s_{1}\right)\ldots \ldots sing\left(s_{n}\right)\right]}^{T}$, $\lambda_{2}\left(s\right)=diag\left(\frac{\lambda_{2i}}{Q_{i}\left(s_{i}\right)}\right)$, and $\rho \left(e\right)=diag\left(\frac{\rho_{i}}{Q_{i}\left(s_{i}\right)}\right)$, for i=1………n.

$sign\left(s_{i}\right)$ is a signum function, which can be defined such that:

(14) $$sign\left(s_{i}\right)=\{\begin{array}{c} 1\;for\;s_{i}>0\\ 0\;for\;s_{i}=0\\ -1\;for\;s_{i}<0 \end{array}$$

The term $Q_{i}\left(s_{i}\right)$ is a new reaching law function designed to reduce the effect of chattering [5]. This function is defined as follows:

(15) $$Q_{i}\left(s_{i}\right)=\delta_{i}+\left(1-\delta_{i}\right)e^{\left(-\alpha_{i}{\left|s_{i}\right|}^{p_{i}}\right)};\;0<\delta_{i}<1\;\mathrm{and}\;\alpha_{i},p_{i}>0$$

The function $Q_{i}\left(s_{i}\right)$ is variable positive, with this variation being bounded between 1 and $\delta_{i}$. Generally, the variation of the control gain is considered an adaptation technique to provide a fast system reaching response. Theoretically, as the system reaches the sliding surface, $\left|s_{i}\right|\to 0$, which means that $\lambda_{1i}/Q_{i}\left(s_{i}\right)\to \lambda_{1i}$ and $\lambda_{2i}/Q_{i}\left(s_{i}\right)\to \lambda_{2i}$. On the other hand, as $\left|s_{i}\right|$ increase, the term $Q_{i}\left(s_{i}\right)$ decreases; hence, $\lambda_{1i}/Q_{i}\left(s_{i}\right)\to \lambda_{1i}/\delta_{i}$ and $\lambda_{2i}/Q_{i}\left(s_{i}\right)\to \lambda_{2i}/\delta_{i}$, bringing about a controller able to quickly reach the sliding surface.

Since h(t) is an uncertainty, this might influence the robot tracking performance. In such case, the control input (13) can be rewritten as:

(16) $$\nu =\lambda_{1}\left(s\right)sign\left(s\right)+\varphi {\dot{z}}^{d}-\varphi Az-\rho \dot{e}-\hat{h}\left(t\right)-\int_{0}^{t}\lambda_{2}\left(s\right)sign\left(s\right)$$

Taking into account the validity of Assumption 5, it becomes possible to use TDE [18] to obtain an estimate of h(t) using Equation (8) as follows:

(17) $$\hat{h}\left(t\right)≅h\left(t-t_{s}\right)={\dot{x}}_{2}\left(t-t_{s}\right)-\nu \left(t-t_{s}\right)$$

where $t_{s}$ is the estimation time delay. It is obvious that for a very small $t_{s}$, $\hat{h}\left(t\right)$ converges to h(t) and in real-time, the smallest possible value of $t_{s}$ is mostly selected to be the sampling time period.

Prior to proving system stability, it is essential to define the uncertain dynamics estimation errors. Utilizing Assumption 4 and Equation (17), those become:

(18) $$\tilde{h}\left(t\right)=h\left(t\right)-\hat{h}\left(t\right)\;=h\left(t\right)-h\left(t-t_{s}\right)\;\leq \sigma \left|x\left(t\right)-x\left(t-t_{s}\right)\right|\leq \sigma \left|t_{s}\right|$$

where σ is the Lipchitz constant.

Substituting control law (16) in system (9), and using the selected sliding surface (12), the proposed modified Super-Twisting control STC for n-subsystems can then be expressed as follows:

(19) $$\{\begin{array}{l} {\dot{s}}_{i}=\frac{-\lambda_{1i}}{Q_{i}\left(s_{i}\right)}{\left(\left|s_{i}\right|\right)}^{\frac{1}{2}}sign\left(s_{i}\right)+\Delta h_{i}+w_{i}\\ {\dot{w}}_{i}=\frac{-\lambda_{2i}}{Q_{i}\left(s_{i}\right)}sign\left(s_{i}\right);\;i=1\ldots .n \end{array}$$

where $\lambda_{1i}>0;\;\lambda_{2i}>0$ are positive constants.

$\Delta h={\left[\Delta h_{1}\ldots .\Delta h_{n}\right]}^{T}$ is the time delay estimation error, subject to the inequality $\Delta h\leq \varrho {\left|s\right|}^{\frac{1}{2}}$ as referenced in [22,23,24,25,26], with ϱ being the uncertainty boundary. Accordingly, Δh can be redefined as follows:

(20) $$\begin{array}{ll} \Delta h & =\sigma \left|x\left(t\right)-x\left(t-t_{s}\right)\right|\leq \sigma \left|t_{s}\right|\\ & \leq \varrho {\left|s\right|}^{\frac{1}{2}} \end{array}$$

For the above inequality to hold, Lipchitz constant σ is chosen to be $\sigma =\frac{1}{{\left(1+\frac{1}{t}\right)}^{2}}>0$, where t is the running time of the desired trajectory and $t_{s}$ is the sampling time (refer to Appendix A.1 for justification).

**Proof.**  To ensure system convergence, consider the following quadratic Lyapunov function:

(21) $$V=\gamma^{T}R\gamma$$

where $\gamma ={\left[\gamma_{1i},\gamma_{2i}\right]}^{T}$, $\gamma_{1i}={\left(\left|s_{i}\right|\right)}^{\frac{1}{2}}sign\left(s_{i}\right),\;\mathrm{and}\;\gamma_{2i}=w_{i}$. The Lyapunov function (21) is chosen to be continuous but not differentiable at $s_{i}=0$ [12]. It is positive definite and radially bounded by choosing an appropriate matrix $R\in {\mathbb{R}}^{2\times 2}$ such that:

$$R=\left[\begin{array}{cc} \frac{1}{2}\lambda_{1i}^{2}+2\lambda_{2i} & \frac{-1}{2}\lambda_{1i}\\ \frac{-1}{2}\lambda_{1i} & 1 \end{array}\right]$$

with

(22) $$\alpha_{min}\left\{R\right\}{\left\|\gamma \right\|}^{2}\leq V\leq \alpha_{max}\left\{R\right\}{\left\|\gamma \right\|}^{2}$$

where $\alpha_{min}\left\{R\right\}\;\mathrm{and}\;\alpha_{max}\left\{R\right\}$ are the minimum and maximum eigenvalues of {R}, while ${\left\|\gamma \right\|}^{2}$ is the Euclidian norm of γ. Taking the derivative of the Lyapunov function (21) gives:

(23) $$\dot{V}={\dot{\gamma }}^{T}R\gamma +\gamma^{T}R\dot{\gamma }$$

The time derivative of γ can be defined as follows:

(24) $$\{\begin{array}{l} {\dot{\gamma }}_{1i}=\frac{1}{2{\left|s_{i}\right|}^{\frac{1}{2}}}{\dot{s}}_{i}\\ {\dot{\gamma }}_{2i}={\dot{w}}_{i} \end{array}$$

Using Equations (19) and (24), $\dot{\gamma }$ can be rewritten in matrix form, with $\gamma_{1i}={\left|s_{i}\right|}^{\frac{1}{2}}$, as follows:

(25) $$\dot{\gamma }=\frac{1}{{\left|s_{i}\right|}^{\frac{1}{2}}}\left[\begin{array}{cc} \frac{-\lambda_{1i}}{2Q_{i}\left(s_{i}\right)} & \frac{1}{2}\\ \frac{-\lambda_{2i}}{Q_{i}\left(s_{i}\right)} & 0 \end{array}\right]\left[\begin{array}{c} \gamma_{1i}\\ \gamma_{2i} \end{array}\right]+\frac{1}{{\left|s_{i}\right|}^{\frac{1}{2}}}\left[\begin{array}{c} \frac{1}{2}\\ 0 \end{array}\right]\Delta h_{i}$$

In the stability analysis, based on Equation (15), the function $Q_{i}\left(s_{i}\right)$ always fulfills the following inequality: $0<Q_{i}\left(s_{i}\right)\leq 1$. Let us assume that $Q_{i}\left(s_{i}\right)=1$; hence, the equation above can be rewritten in the form:

(26) $$\dot{\gamma }=\frac{1}{{\left|s_{i}\right|}^{\frac{1}{2}}}\left(A_{s}\gamma +B_{s}\Delta h\right)$$

where $A_{s}=\left[\begin{array}{cc} \frac{-\lambda_{1i}}{2} & \frac{1}{2}\\ -\lambda_{2i} & 0 \end{array}\right]\;\mathrm{and}\;B_{s}=\left[\begin{array}{c} \frac{1}{2}\\ 0 \end{array}\right]$.

Substituting Equation (26) into (23), the following is obtained:

(27) $$\dot{V}=\frac{1}{{\left|s_{i}\right|}^{\frac{1}{2}}}\gamma^{T}\left(A_{s}^{T}R+RA_{s}\right)\gamma +\frac{2}{{\left|s_{i}\right|}^{\frac{1}{2}}}\Delta h_{i}B_{s}^{T}R\gamma$$

Thereafter, substituting Equation (20) into (27):

(28) $$\dot{V}=\frac{1}{{\left|s_{i}\right|}^{\frac{1}{2}}}\gamma^{T}\left(A_{s}^{T}R+RA_{s}\right)\gamma +\frac{2}{{\left|s_{i}\right|}^{\frac{1}{2}}}\sigma_{i}t_{s}B_{s}^{T}R\gamma$$

Since the inequality $2B_{s}^{T}R\gamma \leq \gamma^{T}M\gamma$ is valid, Equation (28) can be reformulated as:

(29) $$\begin{array}{l} \dot{V}\leq \frac{1}{{\left|s_{i}\right|}^{\frac{1}{2}}}\gamma^{T}\left(A_{s}^{T}R+RA_{s}\right)\gamma +\frac{1}{{\left|s_{i}\right|}^{\frac{1}{2}}}\sigma_{i}t_{s}\gamma^{T}M\gamma \\ \;\leq \frac{1}{{\left|s_{i}\right|}^{\frac{1}{2}}}\gamma^{T}\left(A_{s}^{T}R+RA_{s}+\sigma_{i}t_{s}M\right)\gamma \end{array}$$

where $M=\left[\begin{array}{cc} \frac{1}{2}\lambda_{1i}^{2}+2\lambda_{2i} & \frac{-1}{4}\lambda_{1i}\\ \frac{-1}{4}\lambda_{1i} & 0 \end{array}\right]$.

Equation (29) can be rewritten as:

(30) $$\dot{V}\leq \frac{1}{{\left|s_{i}\right|}^{\frac{1}{2}}}\gamma^{T}D\gamma$$

where D is by definition:

(31) $$D=-\left(A_{s}^{T}R+RA_{s}+\sigma_{i}t_{s}M\right)$$

and is calculated by:

$$D=\frac{\lambda_{1i}}{2}\left[\begin{array}{cc} \lambda_{1i}^{2}+2\lambda_{2i}-\sigma_{i}t_{s}\left(\lambda_{1i}+4\frac{\lambda_{2i}}{\lambda_{1i}}\right) & \frac{1}{2}\sigma_{i}t_{s}-\lambda_{1i}\\ \frac{1}{2}\sigma_{i}t_{s}-\lambda_{1i} & 1 \end{array}\right]$$

The function $\dot{V}$ is negative definite. If $\sigma_{i}>0$, $\lambda_{1i}>2\sigma_{i}t_{s},\;\mathrm{and}\;\lambda_{2i}>\frac{\sigma_{i}^{2}t_{s}^{2}\lambda_{1i}}{8\lambda_{1i}-16\sigma_{i}t_{s}}$, this will ensure that det(D)>0 [12]. Under such conditions, matrix D is positive and symmetric. In such case, Equation (29) can be rewritten as:

(32) $$\dot{V}\leq \frac{-1}{{\left|s_{i}\right|}^{\frac{1}{2}}}\alpha_{\min}\left\{D\right\}{\left\|\gamma \right\|}_{2}^{2}$$

where $\alpha_{min}\left\{D\right\}$ is the minimum eigenvalue of D.

Equation (32) proves that the Lyapunov function is negative. Therefore, the stability of the robot system is proved.

It is now essential to define the finite-time convergence of the sliding surface. As such, utilizing Equation (22), the following is obtained:

(33) $$\frac{V^{\frac{1}{2}}}{\alpha_{max}^{\frac{1}{2}}\left\{R\right\}}\leq {\left\|\gamma \right\|}_{2}^{2}\leq \frac{V^{\frac{1}{2}}}{\alpha_{min}^{\frac{1}{2}}\left\{R\right\}}$$

It is straightforward that ${\left|s_{i}\right|}^{\frac{1}{2}}\leq {\left\|\gamma \right\|}_{2}$. Combining this with Equations (32) and (33):

(34) $$\dot{V}\leq \frac{-1}{{\left|s_{i}\right|}^{\frac{1}{2}}}\alpha_{\min}\left\{D\right\}{\left\|\gamma \right\|}_{2}^{2}\;\leq \frac{-1}{{\left\|\gamma \right\|}_{2}}\alpha_{\min}\left\{D\right\}{\left\|\gamma \right\|}_{2}^{2}\;\leq \frac{\alpha_{\min}\left\{D\right\}}{\alpha_{max}^{\frac{1}{2}}\left\{R\right\}}V^{\frac{1}{2}}$$

Accordingly, the maximum convergence time of the sliding surface can be defined as follows:

(35) $$T=\frac{2\alpha_{max}^{\frac{1}{2}}\left\{R\right\}}{\alpha_{\min}\left\{D\right\}}V^{\frac{1}{2}}\left(\gamma \left(0\right)\right)$$

In the case that $0<Q_{i}\left(s_{i}\right)\leq 1$, refer to Remark 1 in Appendix A.2. The global structure of the control system is shown in Figure 1. □

## 3. Experimental Results

### 3.1. Smart Robotic Exoskeleton (SREx) Model

**Figure 2:**
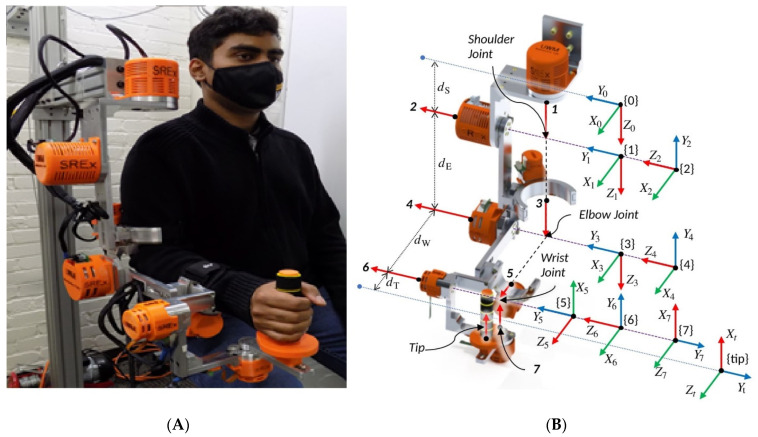
(**A**) SREx maneuvered by a healthy subject. (**B**) Link frame assignment on SREx’s model.

SREx is a redundant (7DOFs) robot of serial manipulator type designed to be worn on the lateral side of the subject’s right upper limb. The seven degrees of freedom (7DOFs) of the exoskeleton makes it a redundant robot capable of achieving several arm configurations in its workspace. This robot is designed to rehabilitate impaired human upper-limbs, as shown in Figure 2. The design of SREx has followed the anatomy and joints of the human upper limb to mimic natural upper limb motion when worn by the subjects during rehabilitation tasks. Its shoulder part comprises three joints: the first two are designed to aid in horizontal and vertical extension/flexion movements, while the third aims to conduct external/internal rotations. The elbow part consists of one joint designed to perform elbow flexion/extension movements. The last part of the upper limb is the wrist, which further consists of three joints: the first is designed to carry out forearm pronation/supination movements; the second and third joints, on the other hand, are designed to perform radial/ulnar deviation and flexion/extension of the wrist, respectively [3,4,5]. The system is equipped with a virtual interface, for which patients and therapists can track the progress of the rehabilitation exercises. The interface can further provide task-oriented exercises in joint space and Cartesian space [28]. The Denavit-Hartenberg (DH) parameters and workspace of the robot are given in Appendix A.3.

### 3.2. Real-Time Setup

**Figure 3:**
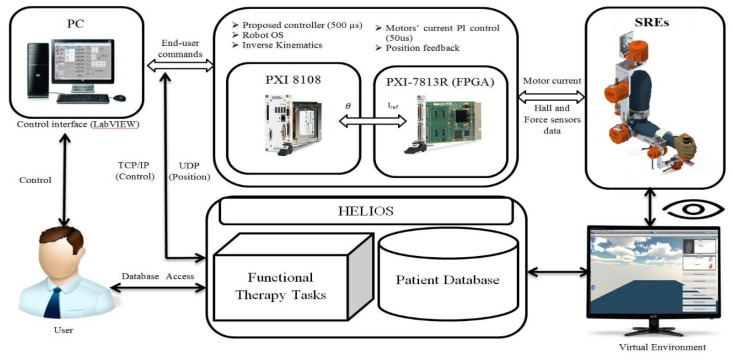
Controller setup.

The exoskeleton robot system architecture is presented in Figure 3. National Instruments, USA PXI was used to control the SREx. As seen in Figure 3, three blocks made the overall experimental setup: The first block is the user interface, used to select and determine controller parameters and define rehabilitation exercise specifications. In addition, it provides the measured robot data, permitting the operator to evaluate the human-exoskeleton system performance accurately. The second block is a PXI-8108 card, where the proposed control was implemented with a sampling time of 1.25 ms. The robot operating system also runs in the PXI processor (Intel Core 2 Duo). The controller outputs are joints torques, which are transformed to currents and then to desired voltages in order to command the motor drivers. Finally, the last block consists of an FPGA (field programmable gate array) that runs with a sampling time of 50 μs. It is utilized to execute two loops concurrently: The first loop holds a simple proportional-integral (PI) action for controlling the motor’s current as a function of the calculated reference current. The second loop is designed to obtain the measured data (position angles) [3,4,29].

Since the control is executed in joint space, to switch the exoskeleton operation into Cartesian space, the inverse Jacobian matrix method is applied. Due to the redundant characteristics of SREx, the inverse kinematics can be obtained using the Jacobian matrix pseudo-inverse, represented as follows [27]:

(36) $$z_{2}=\left(J^{T}{\left(JJ^{T}\right)}^{-1}\right)\dot{X}$$

where $\dot{X}$ is the desired Cartesian velocity, $z_{2}$ is the calculated joint velocity, and J is the robot Jacobian matrix.

In the experimental part, all physical therapy tasks were performed by two subjects (age: 27 ± 4.6 years; height: 170 ± 8.75 cm; weight: 75 ± 18 Kg). The trajectories were generated in Cartesian space in the form of a triangle. In the last section, a comparison analysis against the conventional super-twisting controller (STSMC) proposed in [13] is conducted to confirm the superior efficiency (in terms of trajectory tracking and chattering reduction) and feasibility of the proposed controller. In all subsequent experiments, the initial position of SREx starts with the elbow joint at 90 degrees. Furthermore, controller gains given in Table 1 were experimentally chosen using the trial–error method as follows:

### 3.3. Experiment Results of the Proposed Controller

**Figure 4:**
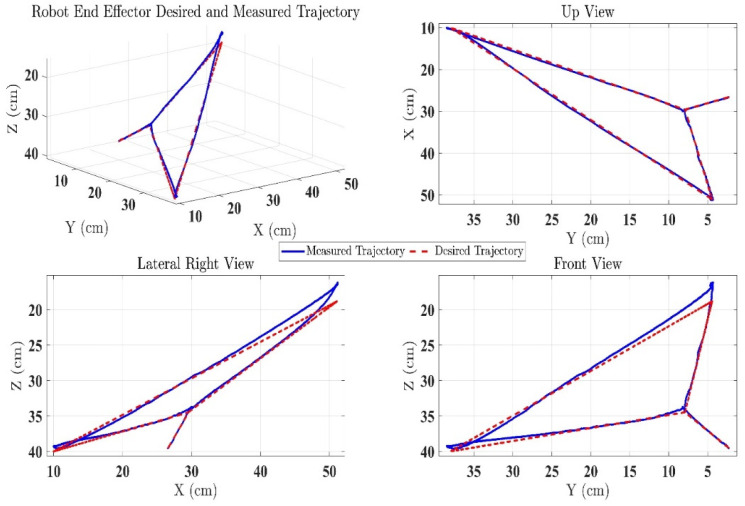
SREx’s trajectory tracking performance in Cartesian space under the proposed controller.

**Figure 5:**
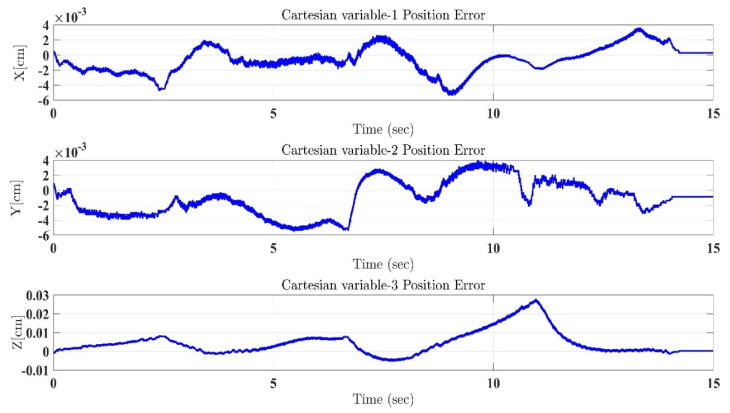
Tracking errors in Cartesian coordinates (X-Y-Z axes) under the proposed controller.

**Figure 6:**
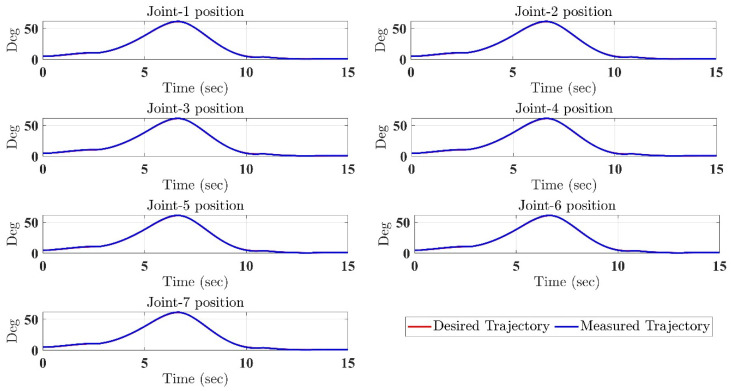
SRex’s trajectory tracking in joint space under the proposed controller (corresponding to Cartesian Trajectory given in Figure 4).

**Figure 7:**
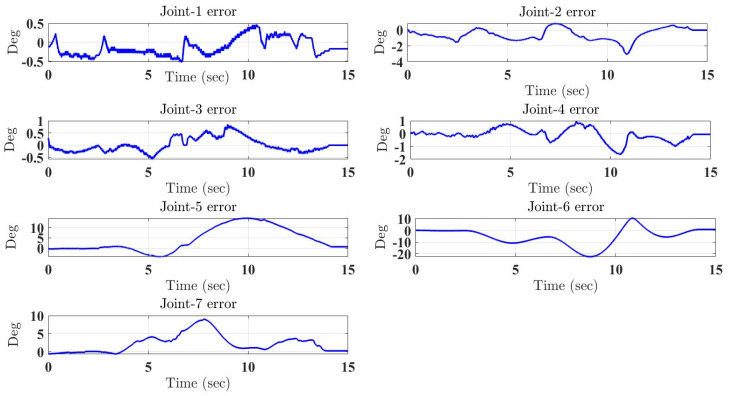
Tracking errors in joint space under the proposed controller.

**Figure 8:**
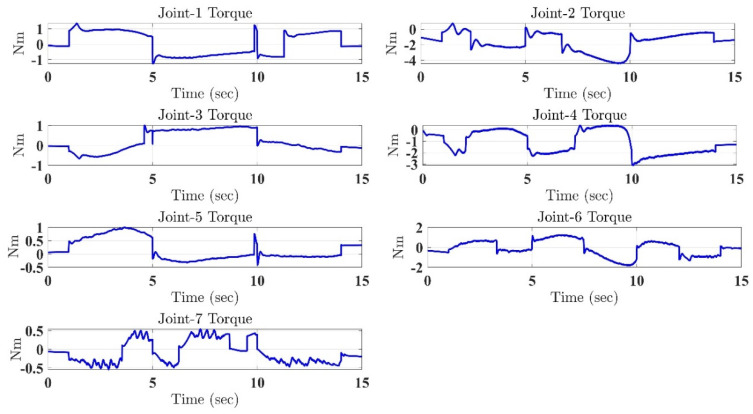
Control inputs under the proposed controller.

In the first test, the proposed exercise consists of a Cartesian triangle trajectory. This exercise was performed by subject-A (age: 30 years; height: 177 cm; weight: 75 kg). Experiment results of the proposed control are given in Figure 4, Figure 5, Figure 6, Figure 7 and Figure 8. Figure 4 presents the trajectory tracking performance of the SREx robot in Cartesian space, and Figure 6 illustrates the results of Joint space. In all the presented results in Figure 4, Figure 5, Figure 6, Figure 7 and Figure 8, the desired/reference trajectories are represented by the red line, and the measured trajectories are represented by the blue line. From the results presented in Figure 4, Figure 5, Figure 6, Figure 7 and Figure 8, it can be safely assumed that the proposed controller provided excellent performance, as described by Cartesian tracking errors in Figure 5 and joint tracking errors in Figure 7, where the controller was capable of maintaining system stability with a maximum discrepancy of 2 to 3 degrees for all joints. Figure 8 represents the control input, which turned out to be smooth (no chattering) for all runs. Besides, one key observation was that the chattering phenomenon did not appear at all. It can be concluded that the controller showed high robustness and precision, whereby even in the presence of unknown parameters, the controller provided highly satisfactory performance.

### 3.4. Experiment Results of the Conventional Super-Twisting Controller (STSMC)

**Figure 9:**
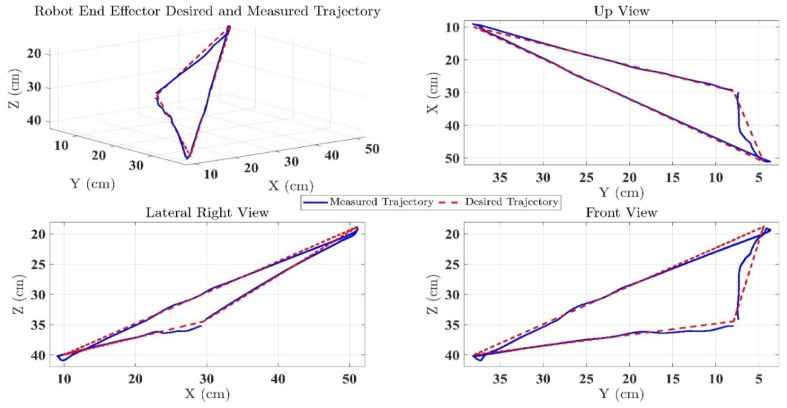
SREx trajectory tracking performance in Cartesian space under the conventional super-twisting controller.

**Figure 10:**
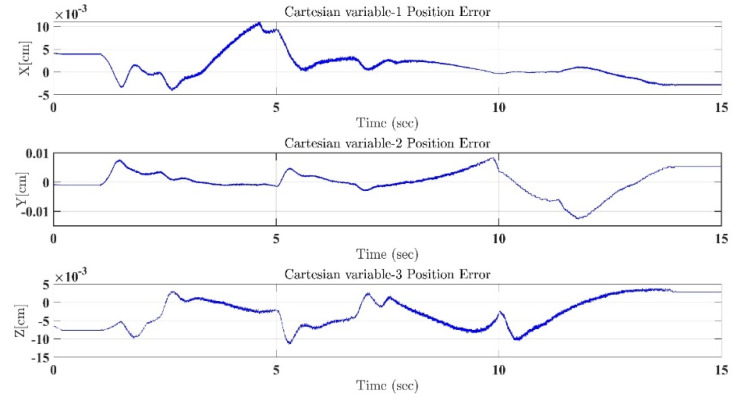
Tracking errors in Cartesian coordinates (X-Y-Z axes) under the conventional super-twisting controller.

**Figure 11:**
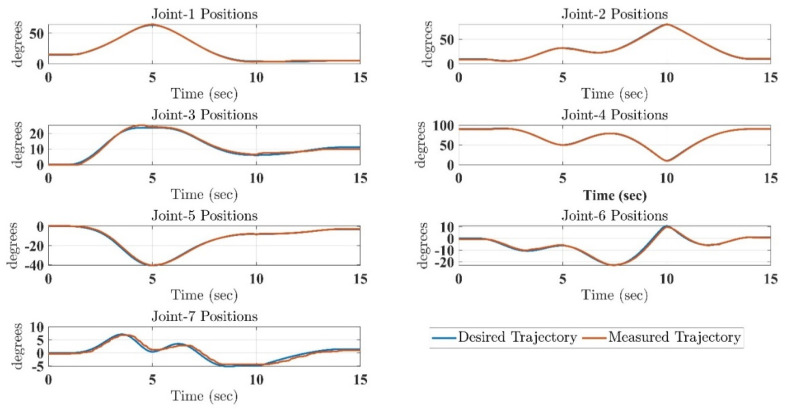
SREx trajectory tracking performance in joint space under the conventional super-twisting controller.

**Figure 12:**
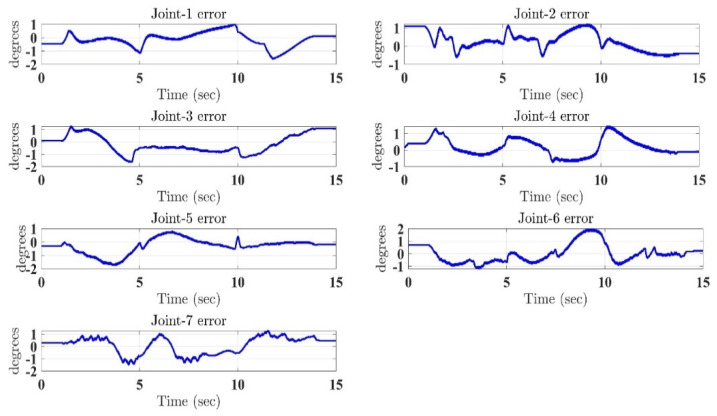
Tracking errors in joint space under the conventional super-twisting controller.

**Figure 13:**
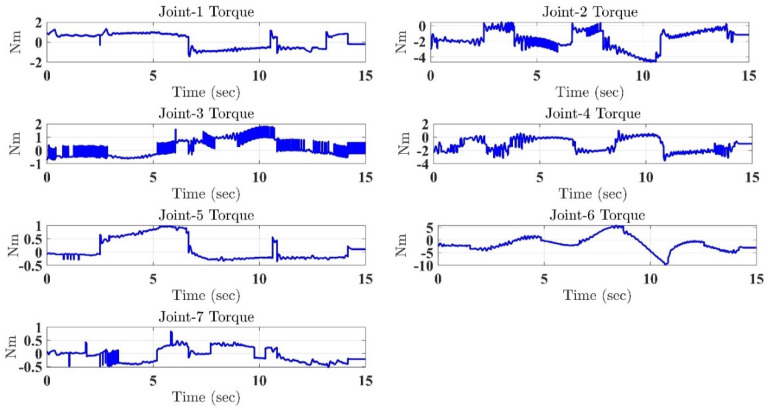
Control inputs under the conventional super-twisting controller.

Similar results corresponding to the same task performed by subject-A were extracted further to explain the robot’s efficiency and outstanding performance. The results of this task, which was completed by subject-B (age: 28 years; height: 176 cm; weight: 80 kg), are illustrated in Figure 9, Figure 10, Figure 11, Figure 12 and Figure 13 under the conventional super-twisting controller (STSMC) proposed in [13]. As described by Figure 9 and Figure 11, it can be readily remarked that for the explained Cartesian and joint movements, respectively, the desired trajectory realistically overlapped on top of the measured trajectory. The controller’s outstanding performance, whereby stability was absolutely maintained across all trajectories, with the small margin of error, is shown by Figure 10 and Figure 12. However, a key observation was that the controller STSMC presented a significant chattering, as shown in Figure 13, compared to the proposed controller that gives a very smooth control input in Figure 8. The smoothness provided by the proposed controller is a result of, firstly, the estimation of the uncertainties, which made the controller design avoid using high gains to reject these uncertainties. Secondly, the adaptive control law (15) helped significantly in reducing the chattering by providing suitable gains based on the position of the system’s trajectory from (close/far) the selected surface.

## 4. Comparative Study

In order to judge whether the proposed control scheme was effective and practical or not, the controller’s performance was compared with the tasks performed by different subjects (different arm weights, physiological conditions, and states of mind), as well as against a conventional super-twisting (STSMC) proposed in [13]. Comparison was carried out by calculating the root mean square (RMS) of each error and the total energy consumed in Cartesian space.

Table 2 clearly suggests that the proposed controller MSTFLTDC provided consistent performance with different subjects, as explained by the small RMS error and torque input values, compared to the STSMC controller. Examining previous similar studies conducted on SREx, the proposed, based on time delay estimation and feedback linearization, presented similar performance to the virtual decomposition controller, but relatively better than both the PID and the Computed Torque Controller [29]. These are extremely desirable results for the next phase of this project: tests with patients suffering from strokes. For example, for the study entitled [30,31] “a clinical trial on upper-limb rehabilitation performed with 15 stroke patients and proving efficiency on assisted rehabilitation task”, the average RMS tracking error, with similar range of motion, was characterized at about 20 degrees (estimated from the different plots in the paper). Using Table 2, the controller tracking error, considering external disturbances and different subject’s physiological characteristics, would account for only 1.4% of that total error. Similar clinical trials’ average RMS error results could be found in other studies [30,32]. This greatly supports the efficiency and suitability of the proposed controller scheme.

## 5. Conclusions

In this research, a robust modified super-twisting controller strategy, using feedback linearization and time delay estimation approaches, was proposed. This allowed a redundant exoskeleton robot to overcome uncertain nonlinear dynamics and external disturbances. In addition, a new reaching law is incorporated with the designed control law to provide fast convergence while reducing the chattering phenomenon. The key advantage of the designed controller is the ability to operate without the need for any information pertaining to the robot’s dynamic model. The results show robust behavior as well as excellent performance tracking for the designed controller, perceived by conducting a rehabilitation task with different subjects. Furthermore, using the Lyapunov theory, system stability was proved in the closed-loop form. Lastly, the experimental results show that the proposed algorithm was efficient and practical. The next step is to investigate the dynamic behavior of SREx while providing therapy to individuals suffering from stroke problems such as spasticity, dystonia, and muscle weakness. The importance of a reliable and robust controller with the characteristics reported in this paper is a key element for this next step to ensure a reliable robot performance as well as to be able to obtain precise data based on subjects’ conditions while keeping robot-related aspects and external disturbances to a negligible margin.

## Figures and Tables

**Table 1:** Controller Parameters.

Constants	Value (*i* = 1:7)
φ	3.2
$\lambda_{1i}$	10
$\lambda_{2i}$	2
$\delta_{i}$	0.5
$\alpha_{i}$	1/2
$p_{i}$	15

**Table 2:** Controller evaluation.

Subjects	Root Mean Square (RMS)			
	MSTFLTDC Controller		STSMC Controller	
	${\left\|e\right\|}_{RMS\;error}$	${\left\|\tau \right\|}_{RMS\;Torque}$	${\left\|e\right\|}_{RMS\;error}$	${\left\|\tau \right\|}_{RMS\;Torque}$
Subject-A	0.0150	2.6908	0.0588	3.2147
Subject-B	0.0129	2.5811	0.0544	3.3827

## Appendix A

### Appendix A.1. Definition of the Lipchitz Constant

In this work, we seek to provide a suitable Time Delay Estimation (TDE) of the dynamic model of the robot in the presence of bounded uncertain dynamics and external perturbations, along unknown boundaries. To address this problem, it is important to validate the following inequality:

(A1) $$\Delta h=\sigma \left|x\left(t\right)-x\left(t-t_{s}\right)\right|\leq \varrho {\left|s\right|}^{\frac{1}{2}}$$

with:

(A2) $$\sigma =\frac{1}{{\left(1+\frac{1}{t}\right)}^{2}}$$

The smallest σ is considered the best Lipchitz constant and is named Lipschitzian maps or contraction mapping [33]. Generally, the Lipschitz constant is chosen between 0 and 1. In such case, the Lipschitz constant, given by Equation (A2), always abides by this condition (0≤σ≤1) when $\underset{t\to 0}{\lim}\sigma =0$ and $\underset{t\to \infty }{\lim}\sigma =1$; hence, the first condition is verified. Next is to verify if this constant abides by the inequality given by Equation (A1). At the initial position, when t=0: $\underset{t\to 0}{\lim}\sigma =0$ and $\underset{t\to 0}{\lim}\left|s\right|=\infty$; thus, at this stage, the inequality holds. If the controller provides good performance, the following becomes true: $\underset{t\to \infty }{\lim}\left|s\right|=0$ while $\underset{t\to \infty }{\lim}\sigma =1$. However, the term $\underset{t\to 0}{\lim}\left|x\left(t\right)-x\left(t-t_{s}\right)\right|\approx 0$; hence, the inequality also holds.

### Appendix A.2. Stability Condition

**Remark** **A1.** *In the case that* $0<Q_{i}\left(s_{i}\right)\leq 1$ *and* $\left|s_{i}\right|$ *increase, the term* $Q_{i}\left(s_{i}\right)$ *decreases; hence,* $\lambda_{1i}/Q_{i}\left(s_{i}\right)\to \lambda_{1i}/\delta_{i}$ *and* $\lambda_{2i}/Q_{i}\left(s_{i}\right)\to \lambda_{2i}/\delta_{i}$*. Let us assume that* $\lambda_{1}=\lambda_{1i}/\delta_{i}$ *and* $\lambda_{1}=\lambda_{2i}/\delta_{i}$*. By using the same procedure from Equation (26) to Equation (31), the condition of stability will be:* $\lambda_{1i}/\delta_{i}>2\sigma_{i}t_{s},\;\mathrm{and}\;\lambda_{2i}/\delta_{i}>\frac{\sigma_{i}^{2}t_{s}^{2}\lambda_{1i}}{8\lambda_{1i}-16\sigma_{i}t_{s}}$*.*

### Appendix A.3. The Workspace and DH-Parameters of SREx

Table A1 presents the workspace of the designed robot.

**Table A1:** Workspace SREx.

Joints	Motion	Workspace
1	Shoulder joint horizontal flexion/extension	0°/140°
2	Shoulder joint vertical flexion/extension	140°/0°
3	Shoulder joint internal/external rotation	85°/75°
4	Elbow joint flexion/extension	120°/0°
5	Forearm joint pronation/supination	85°/85°
6	Wrist joint ulnar/radial deviation	30°/20°
7	Wrist joint flexion/extension	50°/60°

The modified Denavit–Hartenberg (DH) parameters are given in Table A2. These parameters are obtained from the reference frames, as shown in Figure 8, which are used to obtain the homogeneous transformation matrices.

**Table A2:** Modified Denavit–Hartenberg Parameters.

Joint (*i*)	*α*_*i*−1_	*a*_*i*−1_	*d_i_*	*θ_i_*
1	0	0	*d_s_*	*θ_1_*
2	−π/2	0	0	*θ_2_*
3	π/2	0	*d_e_*	*θ_3_*
4	−π/2	0	0	*θ_4_*
5	π/2	0	*d_w_*	*θ_5_*
6	−π/2	0	0	*θ_6_* − π/2
7	−π/2	0	0	*θ_7_*

$\alpha_{i-1}$ is the angle between $Z_{i-1}$ and $Z_{i}$ about $X_{i}$, $d_{i}$ is the distance between $X_{i-1}$ and $X_{i}$ along $Z_{i-1}$, $a_{i-1}$ is the distance between $Z_{i-1}$ and $Z_{i}$ along $X_{i}$, $\theta_{i}$ is the angle between $X_{i-1}$ and $X_{i}$ about $Z_{i-1}$.
